# Isolation and Loneliness: Implications for Health and Well-Being of Older Populations

**DOI:** 10.1093/geroni/igab046.645

**Published:** 2021-12-17

**Authors:** julianne Holt-Lunstad

**Affiliations:** Brigham Young University, Provo, Utah, United States

## Abstract

Evidence suggests social isolation and loneliness are prevalent within the population and may potentially be exacerbated due to the pandemic. Social connections have powerful influences on health and longevity, and lacking social connection qualifies as a risk factor for premature mortality. Evidence from the recent National Academy of Science consensus report on social isolation and loneliness among older adults will be summarized, providing the scope of the health effects, potential mechanisms and risk factors, as well as current gaps in the evidence. Importantly, this evidence points to several implications for solutions across sectors, including medical and healthcare practice and policy.

